# MicroRNAs as Regulators of Immune and Inflammatory Responses: Potential Therapeutic Targets in Diabetic Nephropathy

**DOI:** 10.3389/fcell.2020.618536

**Published:** 2021-01-25

**Authors:** Hong Zhou, Wei-Jian Ni, Xiao-Ming Meng, Li-Qin Tang

**Affiliations:** ^1^Department of Pharmacy, Anhui Provincial Cancer Hospital, The First Affiliated Hospital of USTC, Division of Life Sciences and Medicine, University of Science and Technology of China, Hefei, China; ^2^Inflammation and Immune Mediated Diseases Laboratory of Anhui Province, Anhui Institute of Innovative Drugs, School of Pharmacy, Anhui Medical University, Hefei, China; ^3^Department of Pharmacy, Anhui Provincial Hospital, The First Affiliated Hospital of USTC, Division of Life Sciences and Medicine, University of Science and Technology of China, Hefei, China

**Keywords:** epigenetic regulation, miRNAs, inflammatory, cellular signal transduction, therapeutic target, diabetic nephropathy, immune

## Abstract

Diabetic nephropathy (DN) is the principal cause of end-stage renal disease and results in high morbidity and mortality in patients, causing a large socioeconomic burden. Multiple factors, such as metabolic abnormalities, inflammation, immunoregulation and genetic predisposition, contribute to the pathogenesis of DN, but the exact mechanism is unclear, and the therapeutic strategies are not satisfactory. Accordingly, there is an unmet need for new therapeutic targets and strategies for DN. MicroRNAs (miRNAs) act as major epigenetic mechanisms that regulate gene expression and provide novel insights into our understanding of the molecular and signaling pathways that are associated with various diseases, including DN. Studies in the past decade have shown that different miRNAs affect the progression of DN by modulating different aspects of immune and inflammatory responses. Therefore, in this review, we summarized the pivotal roles of miRNAs in inflammatory and immune processes, with an integrative comprehension of the detailed signaling network. Additionally, we discussed the possibilities and significance of these miRNAs as therapeutic targets in the treatment of DN. This review will facilitate the identification of new therapeutic targets and novel strategies that can be translated into clinical applications for DN treatment.

## Introduction

Diabetes mellitus (DM) is a kind of systemic chronic metabolic disease with hyperglycaemia as the main characteristics, which requires multi-factorial risk-mitigation strategies for long-term medical care. Along with a remarkable rise of living standard, DM is also shaping up to be one of the main contributors to morbidity and mortality on a world scale. Both two types of DM (T1DM and T2DM), particularly T2DM, play a critical role in this worldwide issue due to the influence of related complications (Zheng et al., 2018).

Among those complications, diabetic nephropathy (DN) is becoming the hackneyed and major risk causing cardiovascular mortality and end-stage renal disease (ESRD), and these conditions occur after many years of diabetes (Jiang et al., 2019). Numerous studies have confirmed that multiple mechanisms, such as metabolic abnormalities, haemodynamic changes, inflammatory milieu, oxidative stress and genetic predisposition, continuously contribute to the initiation and progression of DN (Ni et al., 2015). Traditionally, metabolic and haemodynamic factors are the main causes of renal injury in patients with DM and DN. However, recent research has provided compelling evidence showing that chronic inflammation and immunity are associated with the progression of DN, suggesting that immunological and inflammatory mechanisms underpin DN (Gurley et al., 2018). Many research results indicated that both inflammatory factors such as inflammatory cells, cytokines, chemokines and adhesion molecules, and immune mechanisms are all involved in DN pathogenesis, confirming that DN is a chronic inflammatory and immune disorder (Bonacina et al., 2019). However, the precise inflammatory and immunoregulatory mechanisms, therapeutic targets and strategies for DN treatment remain unclear.

Evidence from study demonstrates that many individuals develop DN despite relatively modest hyperglycaemia and hypertension. In addition, some individuals with decades of prolonged hyperglycaemia never develop DN, indicating that there still exist some essential factors at the same time, which will affect the development of DN (Perkins et al., 2019). As research continues, epigenetic modification has become a new research hotspot gradually. Among epigenetic modifications, microRNAs (miRNAs) are important mediators of posttranscriptional feedback control mechanisms that are involved in modulating metabolism, as well as inflammation, which provides unique molecular and cellular insights into the pathophysiology of DN (Li et al., 2018). For example, a recent study reveals that the inhibition of NF-κB-mediated diabetic kidney inflammation and T-bet/Th1-derived renal immune response may be associated with the expression of miR-29b in *db/db* mice (Chen et al., 2014). Furthermore, miR-26a regulates the percentage of Tregs in CD4^+^ T cell cluster and the expression of TGF-β1 by repressing interleukin 6 (IL-6) production, thus having regulation effects on renal immune responses in C57BL/6 mice during diabetic ischaemia-reperfusion injury (Li X. et al., 2019). In addition, kidney-enriched miRNAs, for instance, miR-30 and miR-10 families, as well as miRNAs that are involved in immune responses (such as miR-146a and miR-155), have important roles in modulation of renal function in DN (Lin et al., 2015). Additionally, a recent study shows a high expression of miR-146a and miR-155 in patients and animal model of DN, contributing to the activation of inflammatory pathways, the occurrence of glomerular endothelial inflammation and injury (Huang et al., 2014). The roles of various miRNAs in regulating diabetic renal function by modulating the immune and inflammatory processes are listed in Table 1. For a comprehensive review, a thorough analysis of the literature by consulting resources that are available in the PubMed database through the MESH search headings [(“diabetic nephropathy” OR “diabetic kidney” OR “diabetic renal”) AND (miR OR miRNA OR microRNA) AND (immune OR inflammation OR inflammatory) OR (epigenetics OR ncRNA OR non-coding RNA)] was carried out in addition to a manual search of the reference lists of review articles to find more eligible studies.

**Table 1:** List of miRNAs involved in modulating the immune and inflammatory processes in diabetic nephropathy.

**miRNA**	**Study** **phase**	**Species**	**Expression** **patterns in DN**	**Target gene**	**Biological** **functional**	**References**
miR-146a	Preclinical	C57BL6 mice	Down-regulated	IL-1 receptor-associated kinase 1 (IRAK1)/TNF receptor-associated factor 6 (TRAF6) gene	NF-κB activation, pro-inflammatory cytokines (IL-1, IL-6, and IL-18) ↑, M1 macrophage↑, M2 macrophages↓ inflammation↑	Wu et al., 2019
miR-21	Clinical study	SV129 mice Human	Up-regulated	Cell division cycle 25a (Cdc25a)/cyclin dependent kinase 6 (Cdk6) gene	M2 macrophage↓, M1 macrophage infiltration↑, inflammation↑	Kolling et al., 2017
miR-19b-3p	Preclinical	BALB/c mice C57BL/6J mice	Up-regulated	Suppressor of cytokine signaling-1 (SOCS-1) gene	M1 phenotype polarization, tubulointerstitial inflammation↑	Lv et al., 2019
miR-23a	Preclinical	C57BL/6 mice	Up-regulated	Ubiquitin editor A20/interferon regulatory factor 1 gene	M1 macrophage activation, tubulointerstitial inflammation↑	Li Z. L. et al., 2019
miR-802	Clinical study	C57BL/6J mice Human	Up-regulated	NF-κB-repressing factor (NRF) gene	NF-κB activation, IL-8↓, M1 macrophage activation, inflammation↑	Sun D. et al., 2019
miR-150	Preclinical	C57/BL6 mice	Up-regulated	Suppressor of cytokine signaling-1 (SOCS-1) gene	Th17 cells ↑, IL-17A ↑, renal immune response↑	Sang et al., 2016
miR-202-3p	Preclinical	NOD mice	Up-regulated	Chemokine receptor 7 (Ccr7) gene/Cd247 (Cd3 zeta chain) gene	Infiltrating T lymphocytes↑, renal autoimmunity and injury↑	Fornari et al., 2015
miR-29b	Preclinical	*db/db* mice	Down-regulated	Sp1 gene (inflammation) T-bet gene (immune)	NF-κB-driven renal inflammation↑; CD4^+^IFN-γ^+^ Th1 cell infiltration↑, Th1-associated renal immune injury↑	Chen et al., 2014
miR-27a	Preclinical	*db/db* mice	Up-regulated	Nuclear factor erythroid 2-like 2 (Nrf2) gene	Inflammatory cytokines (IFN-γ, TNF-α, MCP-1 and IL-8)↑, diabetic renal inflammatory response↑	Song et al., 2018
miR-455-3p	Preclinical	Sprague-Dawley rat	Down-regulated	Rho-associated coiled coil-containing protein kinase 2 (ROCK2) gene	Inflammatory cytokines (TNF-α, MCP-1, and IL-1β) ↑, diabetic renal inflammatory response↑	Wu et al., 2018
miR-374a	Clinical study	Human	Down-regulated	Monocyte chemoattractant protein-1 (MCP-1) gene	MCP-1↑, diabetic renal inflammatory response↑	Yang et al., 2018
miR-223	Preclinical	BALB/c mice	Up-regulated	Signal transducer and activator of transcription 3 (STAT3) gene	NF-κB activation, inflammatory cytokines (IL-1 and IL-6) ↑, renal inflammatory response↑	Chen et al., 2012
miR-544	Preclinical	*db/db* mice	Down-regulated	Fatty acid synthase (FASN) gene	NF-κB activation, inflammatory cytokines (IL-1, IL-6, and TNF-α) ↑, renal inflammatory response↑	Sun T. et al., 2019
miR-346	Preclinical	*db/db* mice	Down-regulated	Bruton's tyrosine kinase (BTK) gene; Drosophila mothers against decapentaplegic 3/4 (SMAD3/4) gene	Inflammatory cytokine mRNAs (IL-18 and TNF-α) stabilization and secretion↑, renal inflammatory response↑	Zhang et al., 2015
miR-20b	Clinical study	Human	Down-regulated	Kruppel-like Factor 10 (KLF10) gene	Inflammatory cytokine IL-18↑, renal inflammatory response↑	Zitman-Gal et al., 2014
miR-130a	Preclinical	Sprague-Dawley rats	Down-regulated	Phosphatase and tensin homolog (PTEN) gene	Inflammatory cytokine (IL-18, interferon-γ, IL-1 and TNF-α)↑, tubulointerstitial inflammation↑	Gu et al., 2019
miR-379/411 cluster	Preclinical	MPM cell lines	Down-regulated	IL-18 gene	Inflammatory cytokine (IL-18, interferon-γ, IL-1 and TNF-α)↑, tubulointerstitial inflammation↑	Yamamoto et al., 2014
miR-31	Clinical study	Human	Down-regulated	E-selectin gene	Inflammatory cytokines (TNF-α and IL-6) and the adhesion molecule ICAM-1↑, renal inflammation↑	Rovira-Llopis et al., 2018
miR-29c	Preclinical	Human/*db/db* mice	Up-regulated	Tristetraprolin (TTP) gene	Inflammatory cytokine (IL-6 and TNF-α)↑, renal inflammation↑	Long et al., 2011
miR-1908	Clinical study	Human	Down-regulated	TGF-β1 gene	NF-κB activation, inflammatory cytokines (TNF-α and IL-1β) and the adhesion molecule ICAM-1↑, glomerular macrophage secretion and T cell infiltration↑, renal inflammation↑	Xie et al., 2015
miR-141/200a cluster	Preclinical	C57/BL6 mice	Down-regulated	TGF-β2 gene		Wang et al., 2011
miR-451	Preclinical	db/db mice	Down-regulated	Large multifunctional protease 7 (LMP7) gene	NF-κB activation, chemokines, effector molecules of immunity, inflammatory cytokines, and cell adhesion molecules (TNF-a, IL-18, Myd88, and ICAM-1)↑, renal immune and inflammation response↑	Sun et al., 2016
miR-221/222 cluster	Clinical study	Human	Up-regulated	Adipokine gene	Adiponectin expression↓, inflammatory markers (VCAM-1, TNF, TGF-β1)↑, tubular, glomerular, and interstitial inflammation↑	Al-Rawaf, 2019

*Preclinical, Research involving animals and cells; Clinical study, Research involving human specimens*.

From a pathophysiological point of view, miRNAs are involved in immune and inflammatory processes during the process of DN, but the detailed targeting mechanisms have not yet been comprehensively reviewed due to scattered studies. Therefore, this review focused on highlighting the critical functions of miRNAs in the processes of inflammatory and immune in DN, with an integrative comprehension of detailed molecular biological actions and signaling networks. We also discussed the potential and significance of these miRNAs as therapeutic targets in the treatment of DN. This review will facilitate the identification of new therapeutic targets and strategies, and provide clues to promote the transformation from multiple studies to clinical applications for the targeted treatment of DN.

## Biogenesis and Molecular Functions of miRNAs

Research shows that only a tiny percentage of transcripts (~2–3%) have protein-coding capacity, despite ubiquitous transcription in the whole genomes. This creates an interesting issue of whether the vast majority of transcripts that doesn't code for protein are “useless” in transcription or as important materials which contain much genetic information (Costa, 2010). Extensive sequencing studies have demonstrated that more than 80 percent genomic DNA of mammalian can be zealously transcribed and exquisitely modulated, with the great majority reckoned as non-coding RNA (ncRNA) (Sharp, 2009). The types and amounts of ncRNAs vary among species, and coincidentally, researchers discovered that the complexity of organisms is strongly related to the richness of ncRNA transcripts but weakly correlated with protein coding genes, suggesting the potential research value and significance of ncRNAs. Among these, miRNA is one class of ncRNAs that contains ~22 nucleotides with null encoding ability and is primarily involved in the gene posttranscriptional regulation via mediating mRNA degradation and restraining protein translation in cells (Kabekkodu et al., 2018).

**Figure 1:**
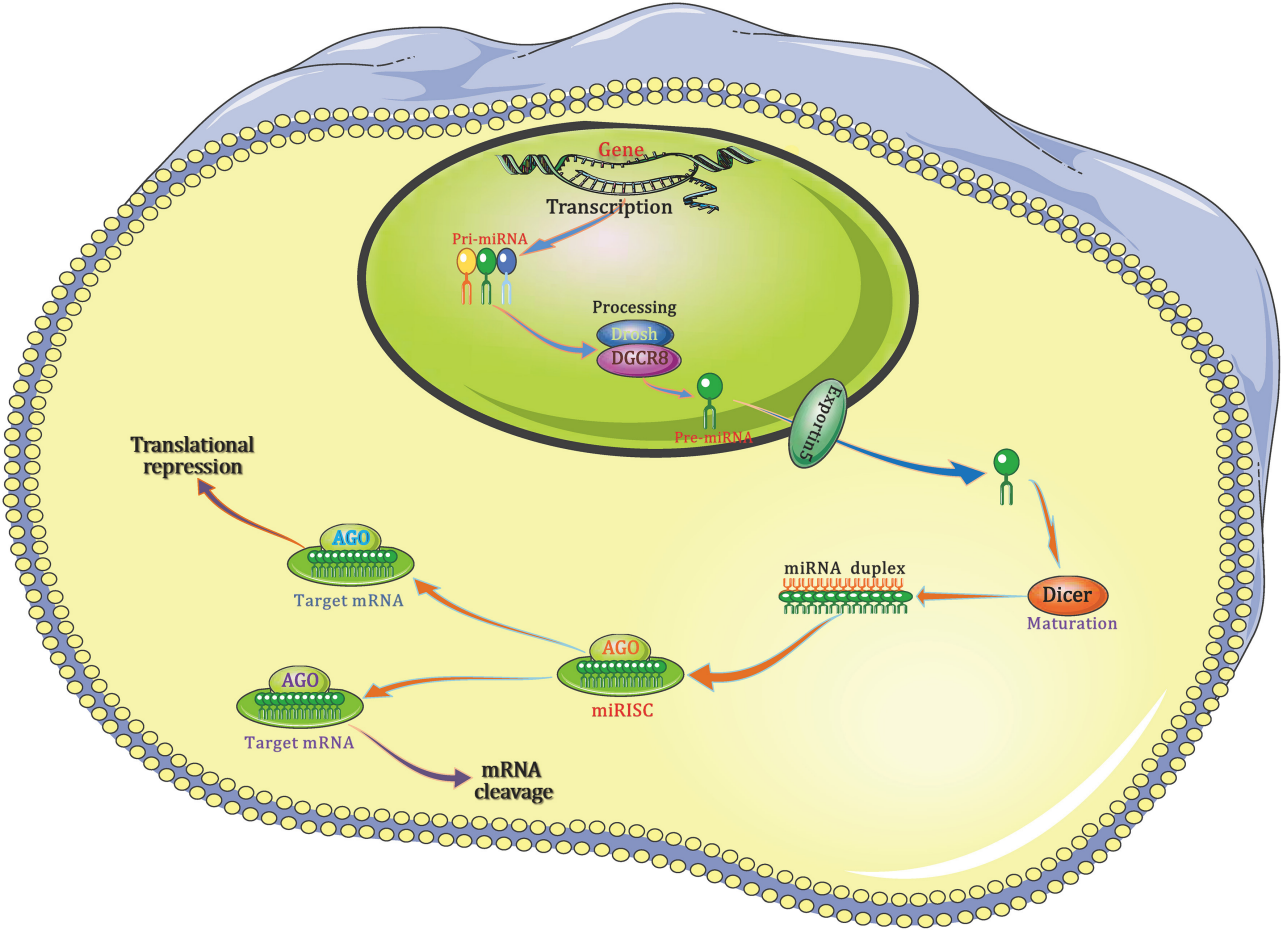
The canonical pathway of miRNA biogenesis. Pri-miRNA is then cleaved by the Drosha/DGCR8 complex to form pre-miRNA, which is exported from the nucleus to the cytoplasm by exportin 5. Once in the cytoplasm, the pre-miRNA is cut into a small, immature dsRNA duplex that contains both the mature miRNA strand and complementary strand by another RNase-III–type endonuclease, Dicer. One strand of the mature miRNA forms a complex with Dicer and the Argonaute protein, also called miRNA-containing RISC, and the miRNA binds to the 3′-UTR of its target mRNA, resulting in target mRNA degradation or translational repression. AGO, Argonaute; DGCR8, DiGeorge syndrome critical region 8; miRISC, miRNA-containing RNA-induced silencing complex; 3′-UTR, 3′-Untranslated regions.

The authoritative path of miRNA biogenesis is considered as a critically regulated and choreographed multi-stage process that starts from nucleus and ends in cytoplasm (Figure 1). Put simply, in nucleus, RNA polymerase II initially transcribes the genes to produce the primary-miRNAs (pri-miRNAs), then, the mature miRNA sequences are embedded in its stem-loop structure. These pri-miRNAs include a poly (A) tail and cap structure, which are distinctive characteristics of type II gene transcript. Then, the non-specific type III ribonuclease Drosha together with the necessary co-factor DGCR8, a protein that contains two double-stranded RNA (dsRNA)-binding domain structures, to generate a protein complex microprocessor (~600 kDa) in the cell nuclei. The formed microprocessor cleaves the aforementioned pri-miRNA into a ~70 base pairs hairpin-shaped RNA known as the precursor-miRNA (pre-miRNA), which consists of a defective loop-stem structure.

After the nuclear processing, pre-miRNAs are transferred to cytoplasm, where the miRNA maturation takes place, through the so-called cytoplasmic cargo transporter named exportin 5 (Exp5). Once it is transported to cytoplasm, pre-miRNA will be cleaved into a kind of imperfect and small dsRNA duplex, which is composed of both the mature miRNA strand and a corresponding complementary strand, by another RNase-III–type endonuclease, Dicer. When combined, the mature miRNA strand together with the Argonaute protein and Dicer to form a complex named miRNA-containing RNA-induced silencing complex (RISC) in which miRNA binds to the 3′-untranslated regions (3′-UTR) of its target mRNA, resulting in the degradation and/or translational repression of mRNA.

## MicroRNAs, Immune Cells, and DN

### miRNAs Modulate Monocyte/Macrophage Function to Affect Inflammation in DN

Monocytes/macrophages have plasticity, can acquire different phenotypes, and exert distinct immune effects under different conditions in the body. Specifically, these cells can be roughly categorized into two distinct phenotypes according their functions, classical M1 macrophages and M2 macrophages. Although both the M1 macrophages and M2 macrophages are monocytes/macrophages, however, they play opposing roles in inflammation (Arora et al., 2018). More specifically, M1 macrophages play significant roles in antigen presentation and inflammatory effects that are generally characterized by increased production of pro-inflammatory cytokines, whereas M2 macrophages release anti-inflammatory cytokines that exert anti-inflammatory effects (Gordon and Martinez, 2010). According to findings obtained from experimental and clinical studies, monocytes/macrophages are considered to be the principal inflammatory cell type that are involved in renal injury and the progression of DN. Additionally, macrophage subtypes are relevant to the circulating monocytes recruitment from vascular space to glomerular tissue, which are closely involved in “renal remodeling” (Tang et al., 2019). Therefore, researchers propose that DN may be alleviated by regulating the infiltration and accumulation of monocytes/macrophages.

**Figure 2:**
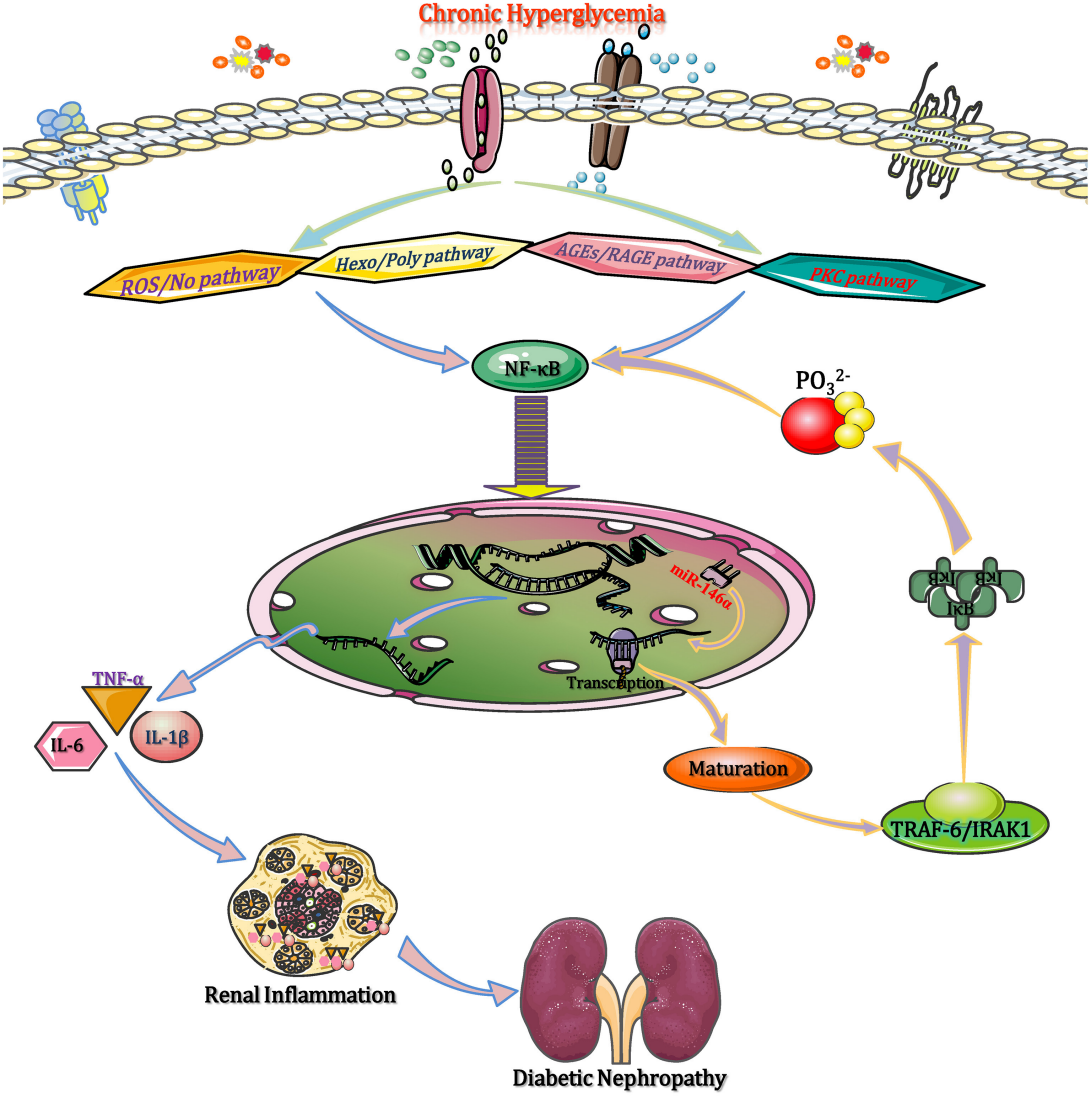
miRNAs modulate monocyte/macrophage function to affect inflammation in DN. In the diabetic state, chronic hyperglycaemia activates NF-κB through multiple intracellular signaling pathways, such as ROS/NO, AGE-RAGE, the Hexo/Poly pathway, and the PKC pathway. Then, NF-κB moves into the nucleus to enhance the expression of several pro-inflammatory genes (TNF-α, IL-1β, IL-6, and IL-18). These pro-inflammatory cytokines contribute to M1 macrophage polarization and NLRP3 inflammasome activation, causing macrophage infiltration and renal inflammation. NF-κB binds to the 3′-UTR of miR-146a to promote its transactivation, and upon processing and maturation, miR-146a enters the cytoplasm and prevents the translation of IRAK1 and TRAF6 mRNAs. Both IRAK1 and TRAF6 activate IKKs, which in turn phosphorylate IκB to release NF-κB. Subsequently, the released NF-κB reduces inflammation to ameliorate diabetic renal inflammation. AGEs, advanced glycation end products; DN, diabetic nephropathy; Hexo/Poly, hexosamines and polyols; IKKs, IκB kinases; IRAK1, interleukin receptor associated kinase 1; NF-κB, nuclear factor kappa-B; NLRP3, NLR family pyrin domain containing 3; PKC, protein kinase C; RAGE, advanced glycation end products and its acceptor; ROS/NO, reactive oxygen species/nitric oxide; TNF-α, tumor necrosis factor-α; TRAF6, tumor necrosis factor receptor associated factor 6.

In the diabetic state, chronic hyperglycaemia activates NF-κB through a range of intracellular signaling pathways, such as reactive oxygen/nitrogen species, advanced glycation end products (AGEs), hexosamines and polyols, as well as protein kinase C (PKC) isoforms (Chen et al., 2018). NF-κB moves into the nucleus to enhance the expression of several pro-inflammatory cytokine genes, such as tumor necrosis factor alpha (TNF-α), IL-1β, IL-6, IL-18, etc. Then, these pro-inflammatory cytokines contribute to the pro-inflammatory shift of macrophages (M1 phenotype) and the activation of NLRP3 inflammasome, causing macrophage infiltration and inflammation in renal tissues, which accelerates the DN progression (Sierra-Mondragon et al., 2018). Moreover, NF-κB activation promotes the transactivation of miR-146a (*via* binding to the 3′-UTR of miR-146a), which, upon processing and maturation, enters the cytoplasm and prevents the translation of TNF receptor associated factor 6 (TRAF6) and interleukin receptor associated kinase (IRAK1) (Wu et al., 2019). These two adapter molecules activate IκB kinases (IKKs), which in turn phosphorylate IκB to release NF-κB from its inhibited state. It has been suggested that miR-146a negatively regulates the activation of NF-κB by reducing IRAK1 and TRAF6 protein expression (Runtsch et al., 2019). Therefore, deficiency of miR-146a can enhance the expression of target genes and result in DN progression by triggering M1 macrophage polarization and activating inflammation, while upregulation of miR-146a suppresses the expression of inflammation related target genes, leading to the pro-inflammatory gene suppression and M1 macrophage activation (Figure 2). Given that, the aforementioned experiment results manifested that miR-146a should be considered as a potential anti-inflammatory miRNA that modulates DN by regulating M1 macrophage activation.

In terms of the role of M2 macrophages in the pathogenesis of DN, at the early stage of infection or injury of diabetic kidney, monocytes differentiate into M1 macrophages to secrete inflammatory cytokines (e.g., IL-1β and TNF-α) and express receptors that are needed for antigen presentation (e.g., MHC II) and pathogen recognition (e.g., TLRs) (Landis et al., 2018). This process is associated with the regulation of miRNAs. Late in the response, the cells differentiate further into M2 macrophages; these cells abrogate inflammatory cytokine production and instead secrete anti-inflammatory cytokines and growth factors (e.g., IL-10 and TGF-β1) (Lee et al., 2011). Under such conditions, the cytokine profile shifts from pro- to anti-inflammatory mediators, with the secretion of IL-10 and IL-1β. TGF-β secreted by M2 macrophages has direct effects on extracellular matrix deposition, as well as favoring the differentiation of proximal tubule epithelial cells into myofibroblasts, which is linked to tubular interstitial fibrosis and crescent formation, eventually accelerating injured renal tissue repair (Gordon, 2003). It was reported that macrophage can enhance the baseline level of PPAR-γ, which is a well-characterized marker of M2 macrophage, and a reduced ratio of nitric oxide synthase/arginase 1 (M1/M2 markers) after knocking out the miR-21. This finding is closely associated with the setting of inflammatory vascular diseases, particularly in DN, because the PPAR-γ activation can reduce the inflammatory response (Kolling et al., 2017). Additionally, in early DN, miR-146a deficiency leads to the M2 markers suppression in macrophages to accelerate DN progression, and the upregulation of miR-146a plays a protective effect by activating M2 macrophage polarization, resulting in suppressed expression of pro-inflammatory and inflammasome-related genes (Bhatt et al., 2016). Therefore, miRNAs affect the inflammatory process in DN by regulating the state of M2 macrophages. In addition, some miRNAs have dual functions. For example, miRNAs (miR-146a, miR-19b-3p, miR-802, miR-23a, and miR-21) are not only capable to block M1 macrophage actions but also improve M2 functions concurrently (Li Z. L. et al., 2019; Lv et al., 2019; Sun D. et al., 2019), which is a beneficial strategy used in DN treatment.

### miRNAs Regulate T Lymphocytes Activities to Mediate Immune and Inflammatory Processes in DN

Compared to the roles of macrophages, less is known about the roles of T lymphocytes in DN. According to authoritative studies, T lymphocytes can not only affect some of the strongest immune/inflammatory responses but also attack a variety of pathogenic organisms or invaders such as viruses and bacteria. During these processes, T lymphocytes can activate or inhibit several important immune system components and thus deregulating/recovering their functions, leading to changes of body immunity (Moon et al., 2012). A study found an increase in T lymphocytes, specifically circulating CD4^+^ T cells, in juxtaglomerular tissues, which resulted in a disturbance in albumin glomerular secretion and decreased renal filtration in T1DM. In addition to peripheral T lymphocytes, both glomerular and interstitial T lymphocytes accumulate in diabetic mice (Gonzalez-Duque et al., 2018).

**Figure 3:**
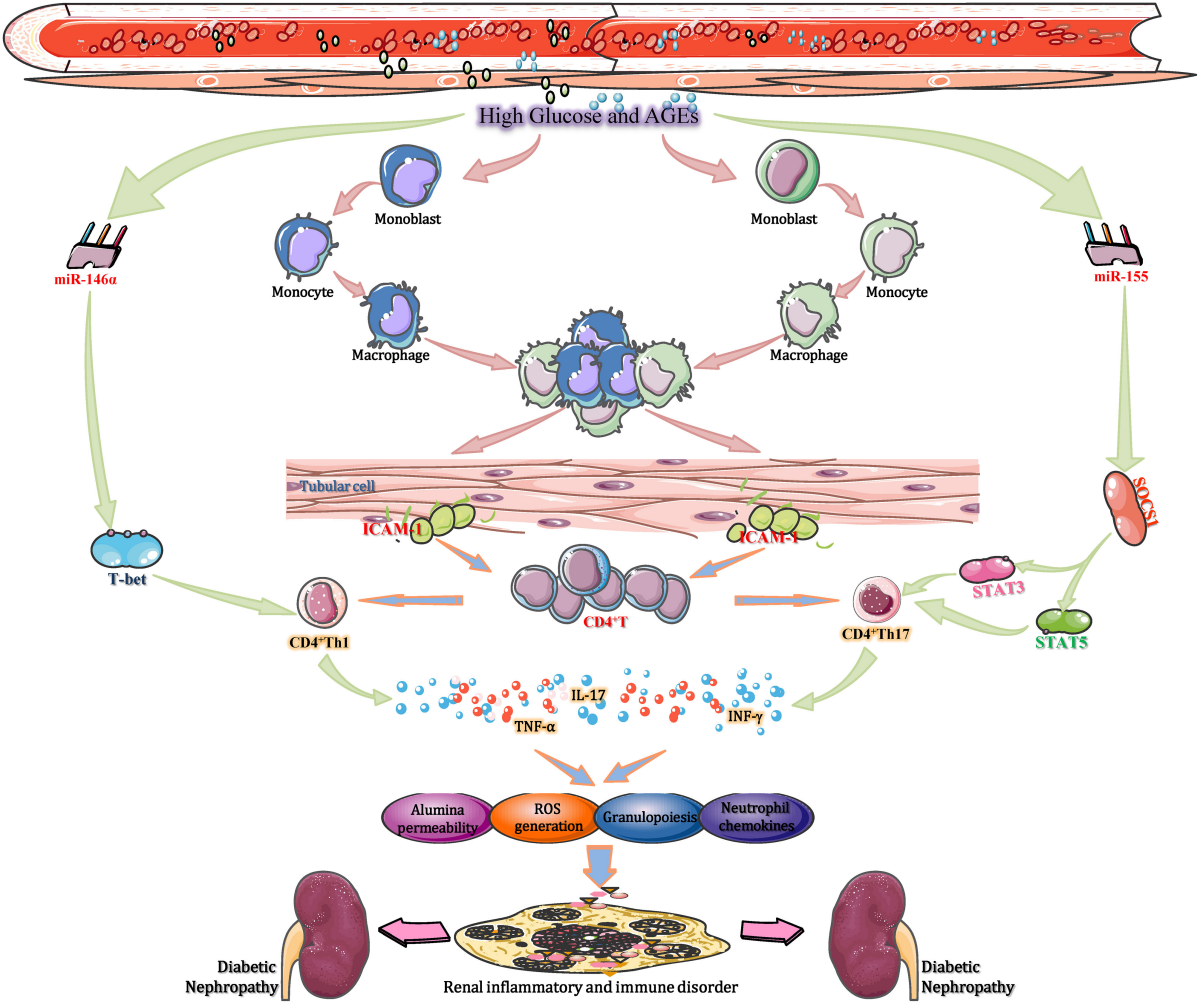
miRNAs regulate T lymphocytes activities to mediate immune and inflammatory processes in DN. During DN progression, miR-155, miR-21, and miR-30b cooperate to promote CD8^+^ T cell activation by repressing SOCS1, DUSP10, and BCL6, respectively. In TIDN, CD3^+^ T cells and pancreas-infiltrating T lymphocytes (PILs) are downregulated by miR-202-3p, which affects the pathogenesis of DN. Moreover, miR155-deficient mice showed a reduced CD4^+^ T cell response to prolong the progression of DN immune disorder. Thus, miRNAs affect DN progression by regulating T lymphocyte-mediated immune functions. BCL6, B-cell chronic lymphocytic leukemia/lymphoma 6; DUSP10, dual specificity phosphatase 10; ICAM-1, intercellular adhesion molecule-1; SOCS1, suppressor of cytokine signaling 1; STAT, signal transducers and activators of transcription; TIDN, type I diabetic nephropathy.

To date, researchers have identified over 100 miRNAs can be considered as potential effect molecules of signal transduction pathways that regulate the functions of multiple immune cells, including macrophages, B lymphocytes, dendritic cells (DCs) and T lymphocytes. Moreover, those enzymes participate in the progression of miRNA biogenesis, such as Drosha, Dicer, and AGO2, also perform important roles in diverse immune cells development processes and subsequent immune responses (Giri et al., 2019). Among many of these studies, several specific miRNAs play key roles in the occurrence, growth, differentiation, as well as effectors/regulatory functions of T cells. For example, miR-150 can negatively regulate the function of CD4^+^ T cell by targeted adjustment of Akt3/Bim pathway in acute graft-vs.-host diseases, for instance, renal transplantation (Sang et al., 2016). Furthermore, three miRNAs including miR-30b, miR-21, and miR-155, work together to facilitate the activation of CD8^+^ T cell through inhibiting the expression of BCL6, DUSP10, and SOCS1, respectively (Salaun et al., 2011). In T1DM, CD3^+^ T cells, and pancreas infiltrating T lymphocytes (PILs) are downregulated by miR-202-3p, which may affect the pathogenesis of DN (Fornari et al., 2015). Moreover, miR155-deficient mice showed a reduced CD4^+^ T cell response to prolong the progression of diabetic renal immunity (Leiss et al., 2017). Thus, miRNAs affect DN progression by regulating T lymphocyte-mediated immune functions (Figure 3). Mechanistically, high glucose or AGEs activate intrarenal macrophage recruitment and in turn increase the intercellular adhesion molecule-1 (ICAM-1) expression in renal tubular cells (Li et al., 2015). The activation of ICAM-1 accelerates intrarenal CD8^+^ and CD4^+^ T cell recruitment, and the recruited T cells, particularly CD4^+^ T cells, infiltrate and accumulate in the tubulointerstitium to develop into CD4^+^ Th1 cells under the stimulation of T-bet, a specific Th1 transcription factor. CD4^+^ Th1 cells produce T cell-related cytokines, such as TNF-α and interferon-γ, in the renal cortex (Demmers et al., 2015). These cytokines not only enhance the local generation of ROS, albumin permeability and inflammatory cell infiltration but also stimulate the excessive T cell-mediated immune response. Over the long term, these progressive anomalies accelerate tubulointerstitial inflammation and local immunological imbalance, ultimately promoting the development of DN (Yang and Mou, 2017). These pathological changes are reversed by overexpression of miR-29b and augmented by knocking down miR-29b in diabetic kidneys in *db/db* mice. The therapeutic effect of miR-29b is attributed to its inhibitory effect on T-bet–dependent Th1-mediated renal injury by binding to the 3′-UTR of T-bet mRNA and thereby inhibiting interferon-γ production (Chen et al., 2014). Another study found that hyperglycaemia enhances the expression of miR-155 in CD4^+^ T cells in renal tissue. Increased miR-155 binding to the 3′-UTR of SOCS1 mRNA to inhibit its transcription and translation led to reduced levels of SOCS1, along with a corresponding increase in signal transducer and activator of transcription 5 (STAT5) and STAT3 phosphorylation (Lin et al., 2015). Then, the percentage of Th17 cells, as well as the expression of IL-17A in CD4^+^ T cells, increased in kidney tissue to mediate neutrophil recruitment and migration through the induction of granulopoiesis and neutrophil chemokines. This results in the gradual exacerbation of inflammatory and immune responses in renal tissues, eventually facilitating DN progression (Yao et al., 2012). It was also demonstrated that knockout of miR-155 reduces the extent of inflammatory and excessive immune responses, which provides a positive impetus for DN treatment (Lu et al., 2009). The aforementioned studies suggest that miRNAs (such as miR-29b and miR-155) affect DN progression by regulating T lymphocyte-mediated immune and inflammatory functions, which are potential therapeutic targets.

## MicroRNAs, Chemokines, and DN

**Figure 4:**
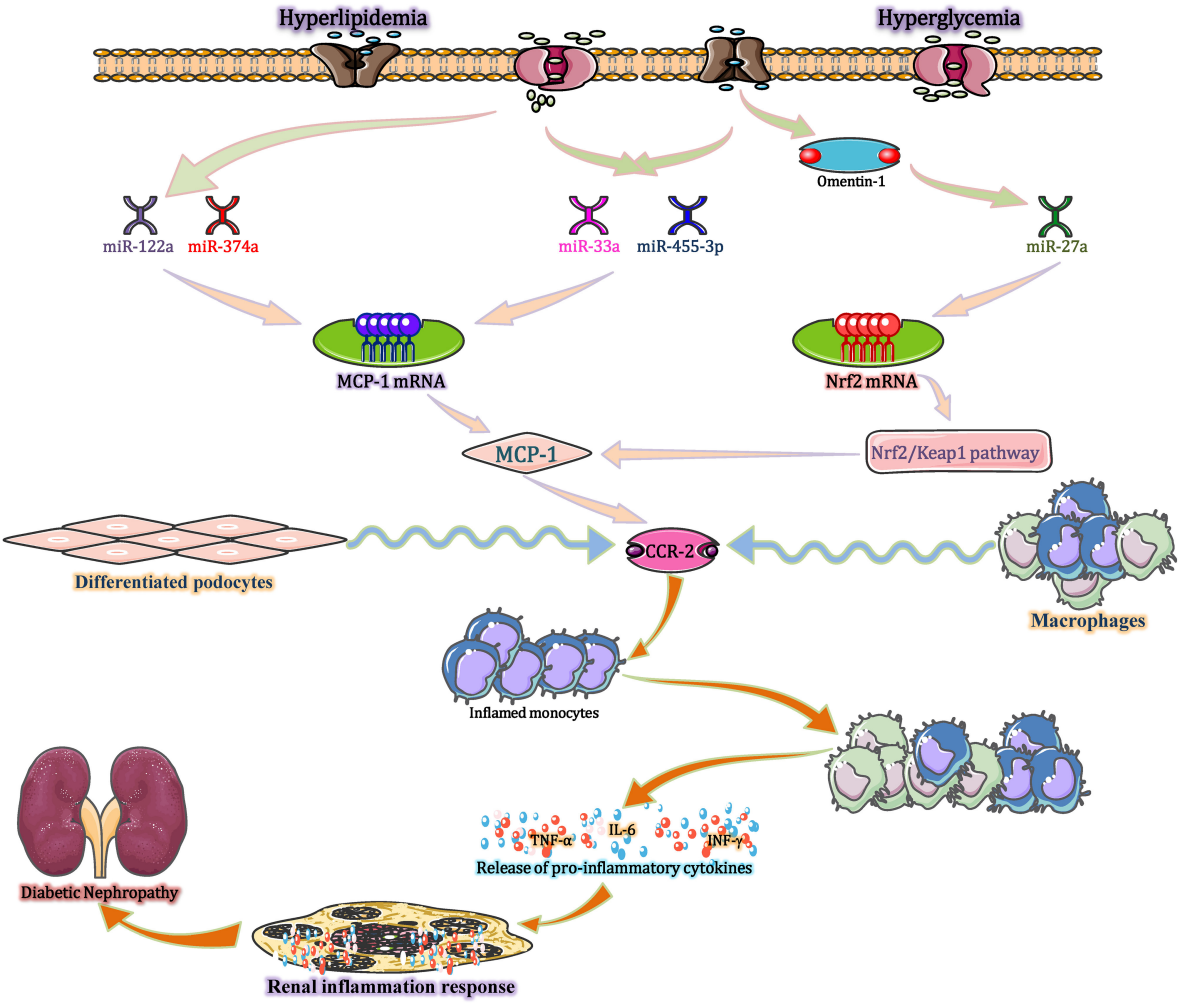
miRNAs modulate inflammatory and immune processes in DN *via* MCP-1/CCL2. Hyperlipidaemia and hyperglycaemia, act as stimulants, not only decreasing the expression of miR-374a and miR-122a but also increasing the expression of miR-33a, miR-455-3p, and miR-27a. These altered miRNAs upregulate MCP-1 expression, and the increased MCP-1 binds to CCR-2 (expressed by monocytes/macrophages and differentiated podocytes) to exert three distinct effects. First, MCP-1 recruits monocytes and precursors into the blood stream. Second, MCP-1 is released at the site of inflammation and stored in the local glycocalyx, thereby forming a chemokine gradient and recruiting circulating monocytes into inflamed kidney tissues. Third, locally produced MCP-1 induces monocyte/macrophage differentiation and inflammatory cytokine expression in the whole kidney. CCL2, chemokine (C-C motif) ligand 2; CCR-2, chemokine receptors-2; MCP-1, monocyte chemo-attractant protein-1; Nrf2, nuclear factor erythroid 2-related factor 2.

Chemokines are small (5–20 kDa) heparin-binding proteins that constitute a large family of similar peptides with high homology among members. At present, more than 50 human chemokines have been identified. Chemokines, such as monocyte chemoattractant protein-1 (MCP-1), fractalkine and interferon-gamma inducible protein (IP-10), are often thought of as important participants to recruit specific inflammatory cell subpopulations into renal compartments, thus mediating the kidney inflammatory reactions and immune responses. Research shows that these molecules are existing in each stage of kidney injury (Ruster and Wolf, 2008). Study further indicates that MCP-1/CCL2 performs a vital role in the recruitment of monocyte/macrophage in DN animal model, as well as renal biopsies from T1DM and T2DM patients (Ruster and Wolf, 2008). The production and activation of MCP-1/CCL2 is regulated by several miRNAs under DN conditions (Kato et al., 2013) (Figure 4).

In the diabetic state, hyperlipidaemia and hyperglycaemia act as stimuli, not only decreasing the expression of miR-374a and miR-122a but also increasing the expression of miR-33a, miR-455-3p, and miR-27a (Lim et al., 2016; Song et al., 2018; Wu et al., 2018). These miRNAs upregulate MCP-1 expression, which enhances glomerular macrophage infiltration in both glomerular and periglomerular renal cortex areas, followed by glomerular monocyte recruitment. Mechanistic investigations reveal that high glucose-mediated down regulation of miR-374a reduces its binding to the 3′-UTR of MCP-1 mRNA to restore MCP-1 transcript and protein expression, increasing the level of MCP-1 in disease state (Yang et al., 2018). Intensive research demonstrates that the increased MCP-1 exerts three distinct effects by binding with C-C chemokine receptor 2 (CCR-2) (produced by monocytes/macrophages and differentiated podocytes) (Tarabra et al., 2009). First, MCP-1 recruits monocytes and precursors into the blood stream. The first recruitment step seems to be increased under inflammatory conditions and is most likely mediated by circulating MCP-1. Second, MCP-1 is released at the site of inflammation and stored in the local glycocalyx, thereby forming a chemokine gradient and recruiting circulating monocytes into inflamed kidney tissues. Third, locally produced MCP-1 induces monocytes/macrophages differentiation and inflammatory cytokine formation in the whole kidney (Haller et al., 2016). In addition, increasing evidence shows that MCP-1 plays a role beyond functioning as a chemoattractant and directly influences other cell types in the kidney. For example, exposure of mesangial cells to MCP-1 increases the expression of inflammatory factors, like the intercellular expression of adhesion molecule-1 (ICAM-1) and several interstitial matrix molecules (Giunti et al., 2006). In the renal tubular epithelial cells of human, MCP-1 stimulates the synthesis of ICAM-1 and secretion of IL-6. Furthermore, in podocytes, MCP-1 binds to CCR-2 to induce the cell migration and a markedly decline in both mRNA and protein levels of nephrin (Tarabra et al., 2009). These effects are reversed by the miR-374a mimic and recapitulated by MCP-1 overexpression. Taken together, miR-374a is active and orchestrates the diabetic renal inflammatory response through mobilization, localization, recruitment, and differentiation by directly regulating MCP-1 (Lee et al., 2017). Recent research indicates that omentin-1 can downregulate the expression of miR-27a and then suppress MCP-1 expression by targeting the Nrf2/Keap1 pathway, thereby decreasing the levels of serum creatinine (Scr), blood urea nitrogen (BUN), and urinary microalbumin (UMA), ultimately improving kidney function in patients with T2DM by ameliorating podocyte dysfunction and glomerular pathological changes (Song et al., 2018). Besides, upregulating the miR-455-3p expression by miR-455-3p mimics or miR-455-3p agomir can subsequently decrease ROCK2 mRNA and protein levels and inhibit RhoA/ROCK signaling. These effects reversed high glucose-induced renal cell proliferation and ECM synthesis by inhibiting the expression of MCP-1, eventually preventing the progression of DN (Wu et al., 2018). All the findings indicate that MCP-1 has a significant role in DN pathogenesis and miRNAs including miR-27a, miR-374a, and miR-455-3p should be considered as potential therapeutic targets for the treatment of DN.

## MicroRNAs, Cytokines, and DN

Cytokines, such as interleukins and TNF-α, are a family of low-molecular-weight, soluble proteins that are synthesized by immune and/or non-immune cells derived from varied embryological origins throughout the body. This class of molecules not only is associated with the regulation process of inflammatory and immune but also act as effectors of inflammatory and immune systems through multiple complex avenues (Syed-Ahmed and Narayanan, 2019). Previously, researchers proposed that inflammatory cytokines are implicated in the DN pathogenesis. Subsequent studies confirm the presence of inflammatory cytokines in different kidney inherent cells (mesangial cell, tubular cell, and endothelial cell), and also immune cells (T cell and monocyte/macrophage), which plays vital roles in the nosogenesis of diabetes mellitus and its microvascular complications (e.g., DN) (Sun and Kanwar, 2015). Under the condition of diabetes, alterations in internal environmental factors, such as hyperglycaemia, hyperlipidaemia, hypertonic states, and microbial infection, cause changes in genetic information and affect the expression and regular activities of several miRNAs by intron transcription of host genes (Baker et al., 2017). The abnormal expression of miRNAs in serum and kidney tissues [such as let-7p, miR-140-5p (Li W. et al., 2019), miR-146a, miR-155-3p (Marques-Rocha et al., 2018), miR-29c, miR-31, miR-33a, and miR-451 (Graham et al., 2015)] regulates the mRNA and protein expression of inflammatory cytokines by activating or inhibiting related signal transduction processes, causing the kidney to produce more of cytokines.

### miRNAs Interfere With Inflammation in DN by Regulating Interleukin 1

**Figure 5:**
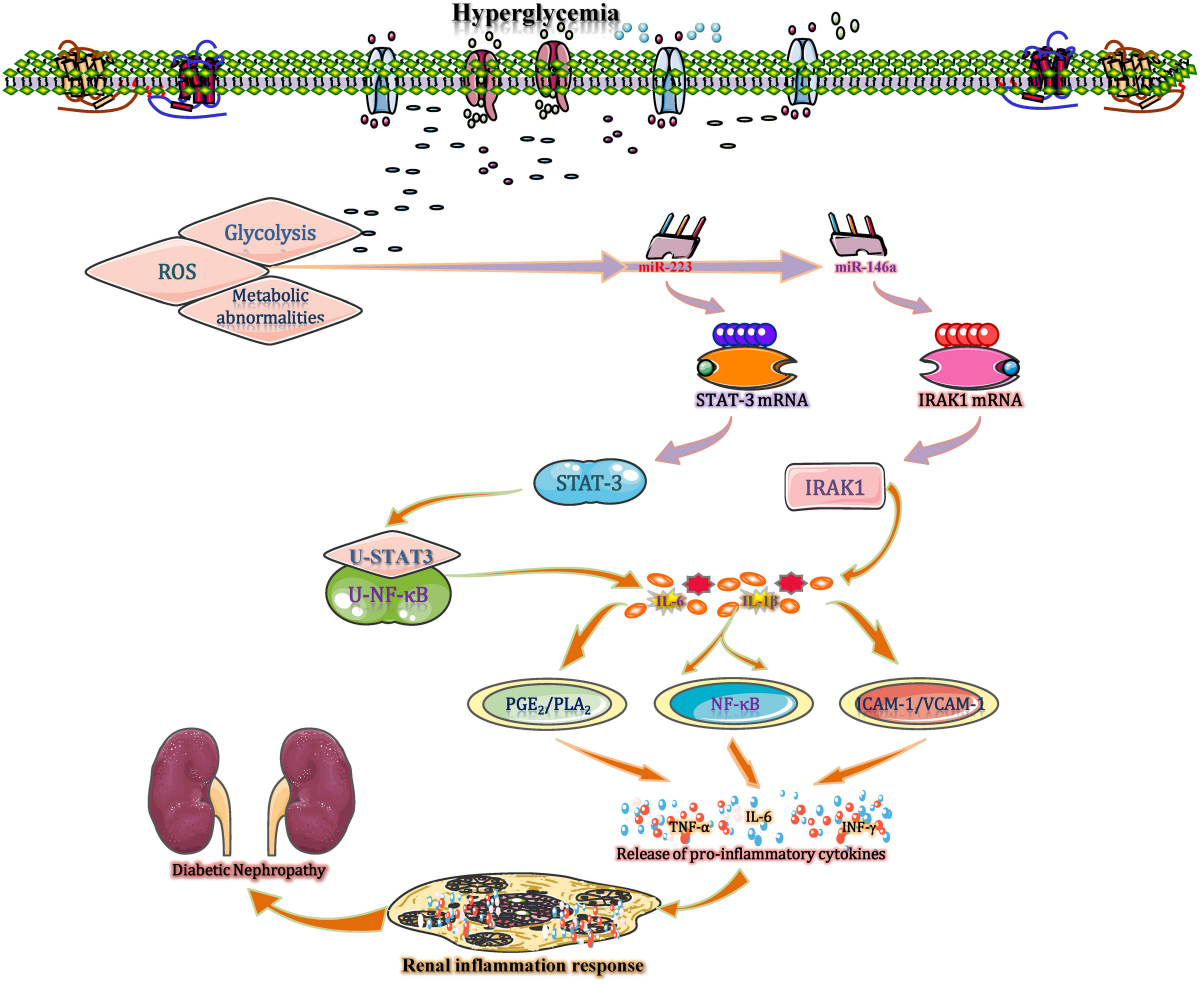
miRNAs interfere with inflammation in DN by regulating interleukin 1. In diabetic kidneys, sustained hyperglycaemia leads to increased glycolysis, ROS production, and the activation of multiple metabolic abnormalities, resulting in a significant increase in miR-223 expression. miR-223 is a non-conserved miRNA that putatively targets the 3′-UTR of STAT3 to regulate its posttranscriptional processing. The un-phosphorylated STAT3 (U-STAT3) combined with un-phosphorylated NF-κB (U-NF-κB) to form a complex and then bind to the κB element site of promoters for transcription of genes, such as IL-1 and IL-6, eventually increase the IL-1 accumulation and amplify the inflammatory response. Moreover, the reduction in miR-146a in kidney tissue increases IL-1 expression with positive feedback regulation and enhances inflammatory responses. In contrast, miR-544 significantly attenuates IL-1 production to ameliorate diabetic renal inflammation by directly targeting fatty acid synthase. PGE2, prostaglandin E2; PLA2, phospholipase A2; U-STAT, unphosphorylated signal transducers and activators of transcription; VCAM-1, vascular cell adhesion molecule-1.

In diabetic kidneys, sustained hyperglycaemia leads to increased glycolysis, ROS, and the activation of multiple metabolic abnormalities, with the latter resulting in the activation of cellular transcriptional machinery (Sheikhansari et al., 2019). In such conditions, the expression of miR-223 is significantly reduced and this miRNA acts as a kind of non-conservative miRNAs that putatively targeting the 3′-UTR of STAT3 to regulate the gene expression (Chen et al., 2012). During this process, an insignificant difference is found in mRNA levels of STAT3 between cells in control group and miR-223 mimic- or antagomir-treated group, indicating that miR-223 inhibits the STAT3′ expression mainly through translational inhibition rather than mRNA degradation. Subsequently, the un-phosphorylated STAT3 (U-STAT3) combined with un-phosphorylated NF-κB (U-NF-κB) to form a complex and then bind to the κB element site of promoters for transcription of genes, such as IL-1 and IL-6, eventually increase the IL-1 accumulation and amplify the inflammatory response in diabetic kidney (Yang et al., 2007; Haneklaus et al., 2013). Moreover, recent studies suggest that variations of tissue-specific and duration-dependent are detected in the course of miR146a expression (Bhatt et al., 2016). During this process, the expression level of miR-146a is substantially below normal level in diabetic kidneys. A reduction of miR-146a reduces its suppressive effect on IL-1 receptor associated kinase (IRAK1) mRNA expression, leading to increased IRAK1 protein expression. High level of IRAK1 contributes to the IL-1-induced NF-κB upregulation, leading to the accumulation of proinflammatory cytokines (IL-1, IL-6, and IL-18) with positive feedback regulation and enhanced inflammatory responses (Chen et al., 2017). IL-1 stimulates several types of renal cells and then increases the levels of E-selectin, VCAM-1, and ICAM-1. When stimulated by IL-1, mesangial cells synthesize prostaglandin E_2_ and release phospholipase A_2_, thereby enhancing inflammatory responses and accelerating the development of renal inflammation (Jia et al., 2011). Additionally, using *db/db* mice, miR-544 is found to significantly attenuate inflammatory cell infiltration and IL-1 production to suppress glomerulosclerosis and inflammation and eventually ameliorate diabetic renal injury by directly targeting fatty acid synthase, suggesting that miR-544 has anti-inflammatory therapeutic potential for DN treatment (Sun T. et al., 2019) (Figure 5). Overall, IL-1 plays a certain role in inflammation in DN, and miRNAs (such as miR-223, 146a, and miR-544) may be potential research targets for DN treatment.

### miRNAs Interfere With Inflammation in DN by Affecting Interleukin 18 Secretion

**Figure 6:**
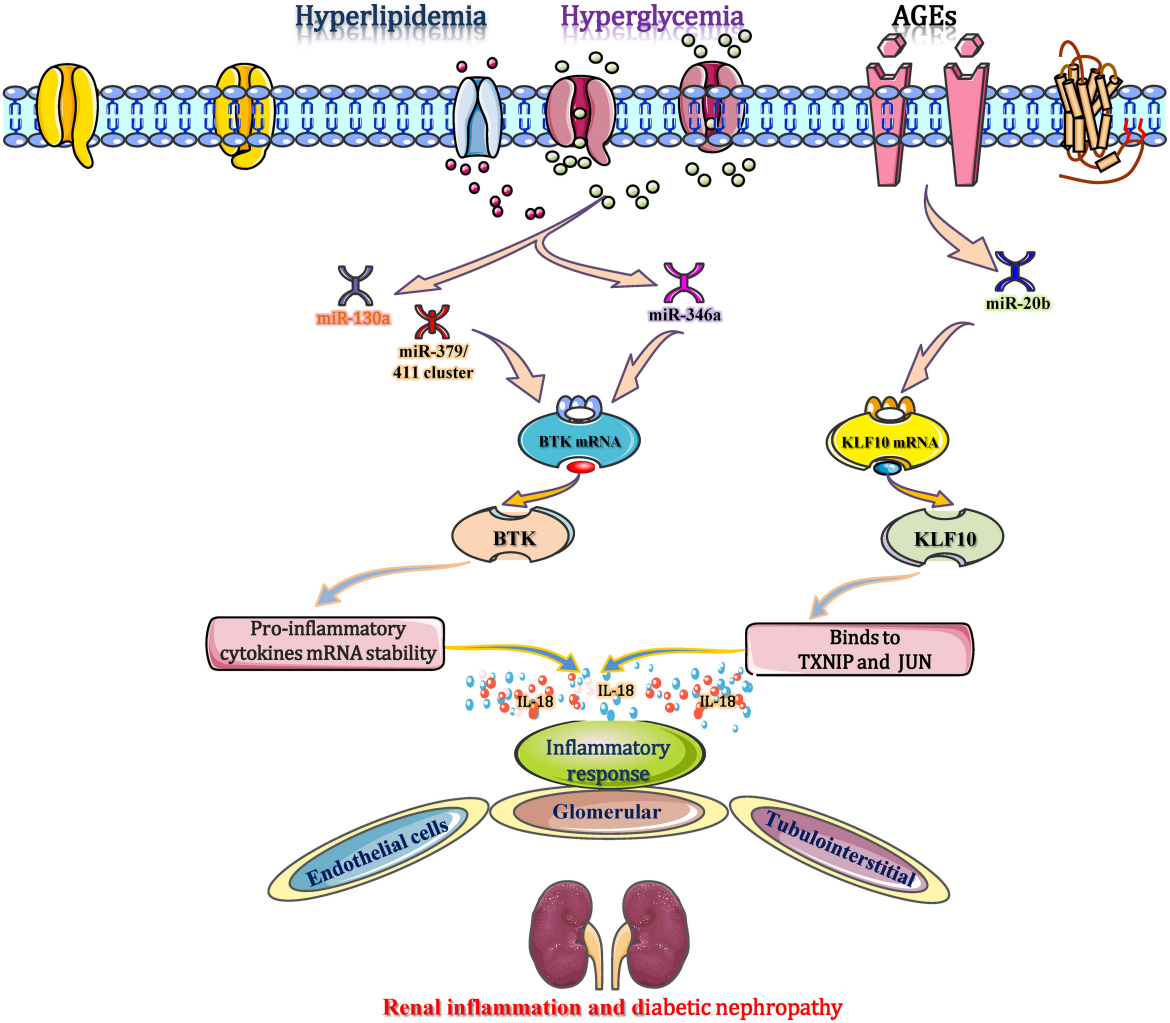
miRNAs interfere with inflammation in DN by affecting interleukin 18 secretion. During the diabetic process, obesity and hyperglycaemia reduce the expression of miR-346 in the renal cortex. miR-346 binds to the 3′-UTR of Bruton's tyrosine kinase (Btk) mRNA to inhibit its transcription, leading to the downregulation of Btk protein. Therefore, the increased Btk stabilizes the mRNA of IL-18 to ensure its secretion and ultimately promotes the inflammatory response in diabetic kidneys. Moreover, high glucose and AGEs down-regulate the expression of miR-15a, miR-20b, and miR-181c. Among them, miR-20b targets the KLF10 gene to promote KLF10 mRNA transcription, which then binds to IL18 to mediate and accelerate the inflammatory response in renal endothelial cells. miR-130a and the miR-379/411 cluster elevate IL-18 to promote the release of IFN-γ, resulting in the production of other inflammatory cytokines, such as ICAM-1, and promoting endothelial cell apoptosis. These alterations are associated with glomerular and tubulointerstitial inflammation and eventually contribute to DN progression. BTK, bruton's tyrosine kinase; KLF10, kruppel-like factor 10; TXNIP, thioredoxin interaction protein.

IL-18 is reported as one of the main prominent cytokines that participate in the inflammatory immune response in DN by polarizing CD4^+^ Th1 cells and inducing the production of inflammatory cytokines, chemokines, and VCAM-1 (Katakami et al., 2007). In C57BL/KsJ-*db/db* mice, obesity and hyperglycaemia accelerated the establishment and progression of DN and reduced the expression of miR-346 in the renal cortex (Zhang et al., 2015). Recently, a study reports that miR-346 binds to the 3′-UTR of Bruton's tyrosine kinase (Btk) mRNA to inhibit the transcription of the Btk gene, leading to downregulation of the Btk protein. These changes suggest that the level of Btk expression is increased in the diabetic setting (Alsaleh et al., 2009). Btk, a 659 amino acid non-receptor tyrosine kinase, is part of Tec family of protein tyrosine kinases, which stabilizes various cytokine mRNAs, such as IL-18 and TNF-α (Chalmers et al., 2016). Btk also stabilizes the mRNA of IL-18 to ensure its secretion and ultimately promotes the inflammatory response in diabetic kidneys. Blocking Btk either chemically (LFM-A13 inhibitor) or by overexpression of miR-346 mimics inhibits the secretion of IL-18, which provides evidence that targeting miR-346 could regulate IL-18 expression as a promising immunomodulating strategy in DN (Alsaleh et al., 2009; Zhang et al., 2015). In diabetic patients with microvascular complications and chronic kidney disease (CKD), high levels of blood/tissue glucose and AGEs down-regulate the expression levels of miR-15a, miR-181C, and miR-20b (Zitman-Gal et al., 2014). Among them, the gradually reduction in miR-20b targets the kruppel-like factor 10 (KLF10) gene to promote KLF10 mRNA transcription, which then binds to IL18 to mediate and accelerate the inflammatory response in renal endothelial cells (Zitman-Gal et al., 2014). In addition, miRNAs (miR-130a and the miR-379/411 cluster) increase IL-18 to promote the release of interferon-γ, resulting in several inflammatory cytokines (such as TNF-α and IL-1) accumulation, ICAM-1 upregulation, and endothelial cell apoptosis (Yamamoto et al., 2014; Gu et al., 2019; Yaribeygi et al., 2019). These alterations are closely and independently associated with glomerular and tubulointerstitial inflammation and eventually contribute to DN progression (Figure 6). Therefore, miRNAs (miR-346, miR-20b, miR-130a, and the miR-379/411 cluster) are potential research targets for intervention in DN-associated inflammation by regulating IL-18 secretion.

### miRNAs Interfere With Inflammation and Immune in DN by Affecting TNF-α Expression

**Figure 7:**
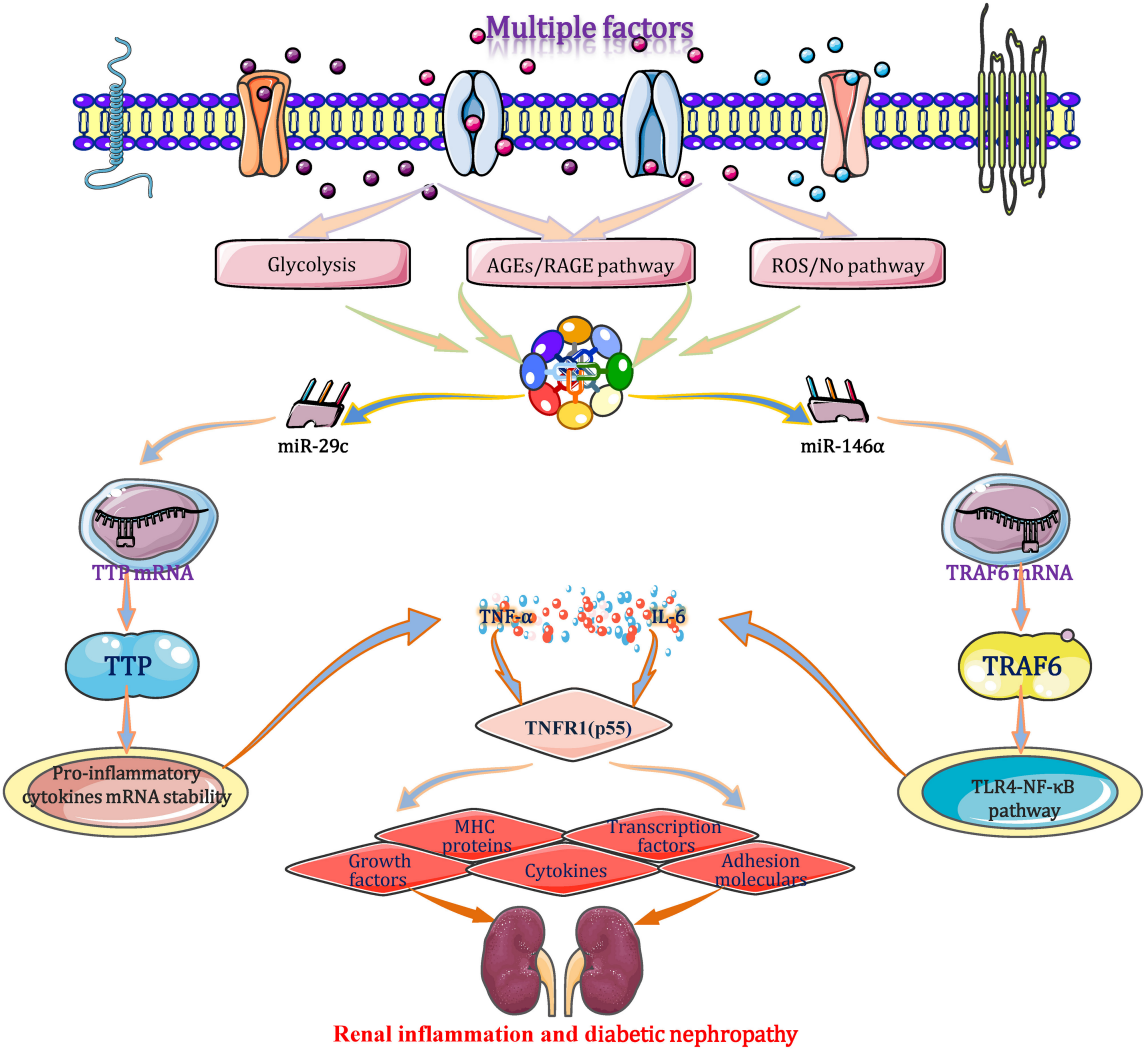
miRNAs interfere with inflammation and immune in DN by affecting TNF-α expression. Under a diabetic state, the expression level of TNF-α is regulated by miRNAs (such as miR-29c, 146a, and miR-31) through several signaling pathways. TNF-α binds to the TNFR1 (p55) receptor, resulting in the production/expression of diverse effectors include adhesion molecules, major histocompatibility complex (MHC) proteins, cytokines, growth factors, transcription factors (TFs) and acute phase proteins, leading to direct renal cell cytotoxicity, apoptosis, intraglomerular haemodynamic alterations, and renal immune/inflammation responses. These alterations play important roles in the courses of inflammatory and immune in DN and may be exploited as potential therapeutic strategies for DN based on the miRNA targets. MHC, major histocompatibility complex; TLR4, toll-like receptor 4; TNFR1, tumor necrosis factor-α receptor 1; TTP, tristetraprolin; TRAF6, tumor necrosis factor receptor associated factor 6.

Under diabetic condition, the expression levels of TNF-α are regulated by miRNAs through several signaling pathways in glomerular and proximal tubular epithelial cells (Bhatt et al., 2016; Rovira-Llopis et al., 2018). A study originally demonstrates that the expression level of the miR-29c show different degrees of upregulation in cultured kidney microvascular endothelial cells, podocytes, and diabetic glomeruli of *db/db* mice (Long et al., 2011). miR-29c binds to the highly conserved 3′-UTR of tristetraprolin (TTP) mRNA and inhibits translation. As an important anti-inflammatory protein, TTP promotes the mRNA degradation and its mRNA instability through combining with the conserved adenosine/uridine-rich element (ARE) that appears at 3′-UTR of mRNA transcripts of cytokines, including TNF-α and IL-6. As a result, both mRNA and protein levels of IL-6 and TNF-α elevate significantly in the end (Guo et al., 2017). TNF-α binds to the TNFR1 (p55) receptor, resulting in the production/expression of diverse effectors include adhesion molecules, major histocompatibility complex (MHC) proteins, cytokines, growth factors, transcription factors (TFs) and acute phase proteins, leading to direct renal cell cytotoxicity, apoptosis, intra-glomerular haemodynamic alterations and kidney inflammation, which play a prominent role in DN pathogenesis (Pavkov et al., 2015). When cells are transfected with a miR-29c mimic or siRNA, the aforementioned signal transduction changes correspondingly. During diabetic progression, miR-146a binds to the 3′-UTR of TRAF6 gene to suppress its transcription and translation, which inhibits the TLR4-mediated NF-κB signaling pathway and restrains the expression and secretion of TNF-α and interleukins. These cytokines negatively regulate the immune response and inflammation to protect against excessive inflammatory activation in multiple tissues (Feng et al., 2017). Beyond that, a result shows a negative correlation between TNF-α and miR-31 (*r* = −0.592; *p* < 0.01) in DN patients, but the mechanism has not been clarified (Rovira-Llopis et al., 2018). Taken together, miRNAs (such as miR-29c, 146a, and miR-31)-mediated TNF-α expression plays a big role during the inflammatory and immune processes of DN, which should be regarded as a potential research target for treating DN (Figure 7).

### miRNAs Regulate TGF-β-Related Renal Inflammation: Crucial Modulators of DN

TGF-β has traditionally been viewed as a key factor in the formation of glomerular fibrosis, glomerulosclerosis and renal fibrosis by regulating the accumulation and degradation of ECM. TGF-β1 has also been regarded as an initial important regulatory factor, whose principal function in inflammation has been extensively studied (Voelker et al., 2017). Recently, *in vitro* and *in vivo* experimental studies of CKD show that TGF-β is positively or negatively regulated by several miRNAs (such as miR-1908, miR-192, and the miR-141/200 cluster) that consequently amplify or reduce the TGF-β signal to further promote or alleviate diabetic renal inflammation (Kato et al., 2011; Xie et al., 2015). These studies suggest that miRNAs are crucial modulators of DN by regulating TGF-β-related renal inflammation.

**Figure 8:**
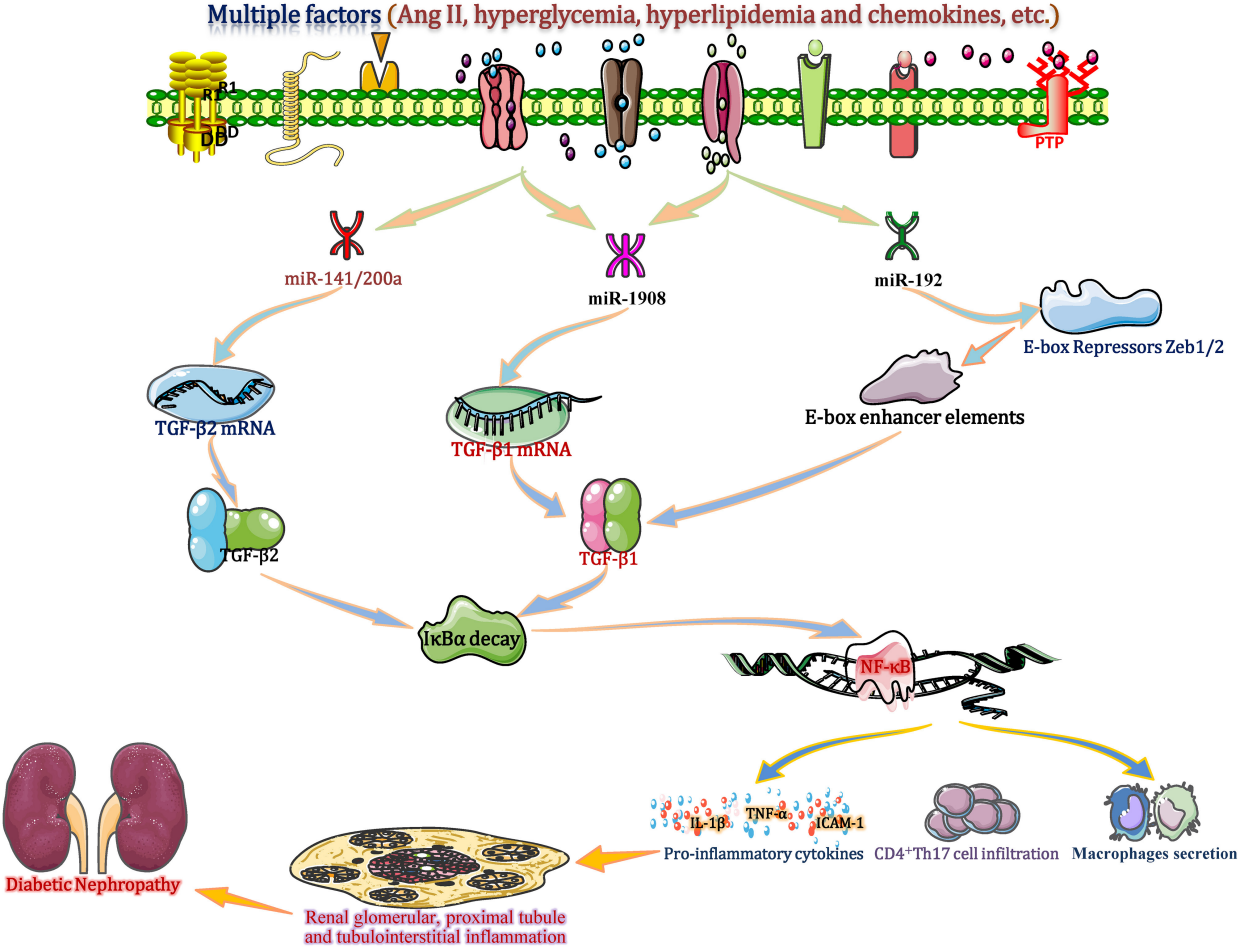
miRNAs regulate TGF-β-related renal inflammation: crucial modulators of DN. Under diabetic conditions, hyperglycaemia, hyperlipidaemia and other factors, such as Ang II and chemokines, cause abnormal changes in several miRNAs, including the miR-1908, miR-192, and the miR-141/200a cluster in renal tissue and interstitial cells. miR-192 leads to increased TGF-β1 expression in end-stage renal disease (ESRD) patients through downregulation of E-box repressor Zeb1/2. Other miRNAs (miR-1908 and miR-141/200a) increase the transcription and translation of the TGF-β1/2 gene by reducing the binding with the 3′-UTR of TGF-β1/2 mRNA, leading to increased TGF-β1/2 expression. TGF-β largely accelerates the decay of IκBα mRNA to promote activation of NF-κB (p50/p65 subunit), which enters the nucleus and induces gene transcription. Then, activated NF-κB increases the accumulation of IL-1β, ICAM-1, and TNF-α and induces glomerular macrophage secretion and CD4^+^ Th17 T cell infiltration in glomerular and tubulointerstitial tissues, eventually promoting renal glomerular, proximal tubule, and tubulointerstitial inflammation and DN progression. ICAM-1, intercellular adhesion molecule-1; IL-18, interleukin 18; TGF-β, transforming growth factor-β; Th17 cell, T helper cell 17; TNF-α, tumor necrosis factor-α; Zeb1/2, zinc-finger E-box binding homeobox 1.

In the setting of diabetes, hyperglycaemia, hyperlipidaemia and other factors (such as Ang II and chemokines) cause abnormal changes in several miRNAs, including miR-1908, miR-192, and the miR-141/200a cluster (Gholaminejad et al., 2018). Among these miRNAs, in renal tissue and interstitial cells, the down-regulated miR-1908 in turn increases the transcription and translation of TGF-β1 gene by reducing its combination with 3′-UTR of TGF-β1 mRNA (Xie et al., 2015). Meantime, the increased miR-192 can enhance the TGF-β1 expression in ESRD patients through downregulation of E-box repressor Zeb1/2 (Kato et al., 2011). Moreover, in a proximal-tubular epithelial cell DN model, decreased miR-141/200a promotes TGF-β2 mRNA translation and protein expression by diminishing miR-141/200a binding to the target site in the 3′-UTR (CAGUGUUA; http://www.targetscan.org) of TGF-β2, resulting in increased expression of TGF-β2 (Wang et al., 2011). Excessive TGF-β largely accelerates IκBα mRNA decay to promote NF-κB/p50/p65 binding to IκBα, which enters the nucleus and induces gene transcription, which in turn induces inflammatory molecular changes (Yang et al., 2019). Then, activated NF-κB increases the accumulation of IL-1β, ICAM-1, and TNF-α and induces glomerular macrophage secretion and CD4^+^ Th17 T cell infiltration in glomerular and tubulointerstitial cells, eventually enhancing proximal tubule and tubulointerstitial inflammation and DN progression (Lan, 2011) (Figure 8). miR-1908, miR-141/200a or miR-192 mimic experiments showed a positive effect on TGF-β1/2 upregulation and inflammatory responses (cytokine production, macrophage secretion and T cell infiltration), suggesting that miR-1908, miR-192, and miR-141/200a are not only biomarkers but also potential mediators of inflammation in DN by regulating the TGF-β-NF-κB pathway (Kato et al., 2011; Xie et al., 2015).

### miRNAs Modulate TF-Related Renal Immune Responses: A Crucial Modulator of DN

NF-κB is recognized as one of the main transcription factors (TFs) involved in DN. In kidneys of diabetic patients and *db/db* mice as well as hyperglycaemia cultured mesangial cells, miR-451 is markedly downregulated. Decreased miR-451 directly alleviates its inhibitory effect on the expression of large multifunctional protease 7 (LMP7) by interacting with the 3′-UTR of LMP7 mRNA. Then, the increased LMP7 promotes NF-κB activation (Sun et al., 2016). Moreover, signals include ligands binding to TNF receptor, T cell receptor, B cell receptor, and TLR–IL-1 receptors can activate a multisubunit IκB kinase complex to phosphorylate the IκB in renal cells (Yang et al., 2019). And then, the phosphorylated IκB comes through a proteasomal degradation, which will facilitate the nuclear translocation of free NF-κB, thus binding to the promoter and enhancer sites, and eventually initiating the transcription process. The above-mentioned actions promote the target genes' transcription to encode inflammatory cytokines, immune effectors, cell adhesion molecules and chemokines, which will perpetuate inflammatory responses and accelerate DN progression (Liang et al., 2018). Furthermore, miR-451 mimics or overexpression in human mesangial cells (HMCs) inhibits the transcription and translation of those target genes, reducing microalbuminuria and blood glucose levels, eventually ameliorating the early progression of DN inflammation (Sun et al., 2016).

In addition to the transcription factor NF-κB, nuclear factor E2-related factor-2 (Nrf2) is another important TF that counteracts the renal oxidative stress and inflammation by regulating its target gene expression (Nezu et al., 2017). In the T2DM model of *db/db* mice, abnormal lipid metabolism and hyperglycaemia can elevate the expression level of miR-27a in kidney (Song et al., 2018). The increased miR-27a directly binds to the 3′-UTR of Nrf2 mRNA to inhibit Nrf2 expression; subsequently, less Nrf2 interacts with the regulatory region of proinflammatory genes and weakens its inhibition of RNA polymerase II recruitment to promote gene transcription. The expression and accumulation of multiple proinflammatory cytokines (TNF-α, IL-8, IFN-γ, and MCP-1) eventually enhance the renal inflammatory response and accelerate DN progression (Ito et al., 2019). These studies suggest a potential effect of miRNAs (miR-451 and miR-27a) in regulating the inflammation through modulating transcription factors during DN, which will lead to the development of novel therapeutic strategies for DN.

## MicroRNAs, Adhesion Molecules, and DN

Adhesion molecules, including ICAM-1 and VCAM-1, are reported to have the ability to promote the interaction between circulating leukocytes and activated endothelium, which are important in the immune response and inflammatory reaction in DN (Nawaz et al., 2019). In diabetic kidneys, sustained glucose decreases the expression of miR-146a in renal tissues and multiple cell types, such as renal tubular cells. Moreover, serum levels of miR-31 are also specifically reduced. The decrease in miR-146a reduces its binding to the 3′-UTR of NADPH oxidase 4 (Nox4) mRNA, thus promoting the protein expression and enhancing the activation of ICAM-1 and VCAM-1 (Wan and Li, 2018). In contrast, another study find a negative correlation between miR-31 and the levels of ICAM-1 and VCAM-1 (*r* = –0.687; *p* < 0.001), suggesting that miR-31 can enhance both the two adhesion molecules (Rovira-Llopis et al., 2018). When ICAM-1 is increased, it mediates the combination of T cells and endothelial cells, which facilitates T cell transmigration into kidney tissues (Anderson et al., 2019). Moreover, increased ICAM-1 and VCAM-1 expression promotes the infiltration and accumulation of monocytes in the kidney and induces monocytes to differentiate into macrophages, further inducing the generation of inflammatory cytokines, including IL-6 and TNF-α (Liu et al., 2015). These actions accelerate renal injury and DN progression by exacerbating inflammation and the immune response. Moreover, overexpression of miR-146a results in the ICAM-1 deficiency, and the homing proportion of CD4^+^ T cells into glomeruli are decreased compared to that of mice that express ICAM-1 (Bhatt et al., 2016).

The negative regulatory role of miRNAs (miR-146a and miR-31) in VCAM-1 and ICAM-1 expression causes renal inflammatory and immune response disorders and accelerates the progression of DN, indicating that miRNAs are potential therapeutic targets in the prevention/treatment of DN.

## MicroRNAs, Adiponectin, and DN

Adiponectin, a kind of endogenous bioactive peptides, is primarily distributed in many areas, including of peritubular capillaries and glomerular endothelium as well as the intrarenal arterioles and arteries in renal tissues. Several studies show that the dysregulation of adiponectin and its receptors can be observed in the progression of multiple diseases, such as hypertension and T2DM, which are the risk factors for the occurrence and development of DN (Panduru et al., 2015). Moreover, an *in vitro* investigation reveals that adiponectin can be secreted from podocytes and tubular epithelial cells, and is mainly eliminated under the conditions of glomerulosclerosis and interstitial inflammation, while it is found in tubular casts (Rutkowski et al., 2017). By inference, adiponectin may prevent the progression of DN through its anti-inflammatory effects on interstitial inflammation and tubular damage.

Under hyperglycaemic and dyslipidaemia conditions, miR-95 and miR-143 expression are positively related with serum adiponectin levels, whereas miR-181a, miR-221, and miR-222 are negatively related to serum adiponectin levels (Al-Rawaf, 2019). These miRNAs regulate the expression of adiponectin mRNA and protein in kidney and circulating blood by interacting with the 3′-UTR of adiponectin mRNA. In the renal cortex, including glomerular, tubulointerstitial and tubular areas, adiponectin binds to its receptors, adiponectin receptor-1 (ADIPOR1) and adiponectin receptor-2 (ADIPOR2), to activate the PPAR-α and AMPK mediated signaling transduction, respectively (Kim et al., 2018). Also, adiponectin receptors display high ceramidase activity, which can be used to transform ceramides to fatty acids and sphingosines. The aforementioned actions subsequently decrease the production of inflammatory markers, such as VCAM-1, MCP-1, TNF-α, and TGF-β1, eventually ameliorating renal tubular and glomerular inflammation (Zhang et al., 2018).

In a T2DM model in C57BL/6J mice, miR-876-3p is a major determinant for the production of adiponectin through targeting the 3′-UTR of adiponectin. Further study shows that lentiviral-mediated miR-876-3p inhibition can increase the production of adiponectin, leading to the amelioration of insulin resistance (IR) and reducing the expression of proinflammatory cytokines, like TNF-α. In contrast, overexpression of miR-876-3p can decrease the expression levels of mRNA and protein of adiponectin and induce the IR and proinflammatory cytokine accumulation (Rajan et al., 2018). These changes indicate that miRNA (miR-95, miR-181a, miR-143, miR-221/222, and miR-876-3p)-mediated adiponectin expression affects the inflammation and may be a potential research target in the progression of DN.

## Clinical Significance of MicroRNAs in DN

At present, several miRNA-based biological agents have progressed into the clinical evaluation phage or even clinical application, such as miRNA-122-related biological agents (LNA-antimiRTM-122, Santaris Pharma) for chronic hepatitis C (Janssen et al., 2013) and miRNA-34-based biological agents (miRNA mimic-MRX34, MIRNA Therapeutics) for liver cancer (Ling et al., 2013). Moreover, many miRNA-associated biological agents (such as the anti-miR-17 oligonucleotide produced by Regulus Therapeutic and the LNA antimiR-155 developed by miRagen Therapeutics) have entered preclinical evaluation (Seto et al., 2018; Lee et al., 2019). These cases bode well for the future use of miRNAs in clinical treatment.

Since their discovery, multiple miRNAs have been studied *in vitro* and *in vivo* in the occurrence and development of DN. Several miRNAs (such as miR-146a, miR-21, and miR-31) regulate the expression of target genes, thus affecting the progression of DN by mediating inflammatory and immune-related signaling pathways, which are listed in Table 1. Many of these miRNAs are upregulated and several are downregulated in the pathological process of DN. Therefore, both up- and downregulation of DN-suppressing and DN-promoting miRNAs can be exploited as therapies for DN in the future. Based on these studies, many potential intervention approaches are proposed to achieve clinical application based on research of the pharmacological inhibition/downregulation of miRNAs through using miRNA inhibitors, anti-miRNA oligonucleotides (AMOs) or miRNA sponges, while upregulation of miRNAs can be accomplished by employing miRNA inducers, miRNA mimics and miRNA-containing exosomes. Therefore, combining intervention methods with miRNA candidates may be a breakthrough in fighting DN by intervening in inflammatory and immune processes. For example, after DN onset in *db/db* mice, delivery of miR-21 knockdown plasmids with ultrasound-microbubble-mediated gene transfer into the diabetic kidneys reduced TNF-α, IL-1β, and MCP-1 in diabetic kidneys, suggesting that miR-21 plays a role in renal inflammation during DN (Zhong et al., 2013). Given this, miR-21 knockdown plasmids by ultrasound-microbubble-mediated gene transfer offer an alternative therapeutic strategy for future clinical evaluation. Moreover, in C57BL/6J mice with induced DN, local treatment with lentivirus particles encoding a miR-802 sponge by ultrasound-microbubbles effectively suppressed the mRNA levels of inflammatory factors, including TNF-α, IL-6, iNOS, and MCP-1, thus blocking macrophage accumulation and reducing the inflammatory response in diabetic kidneys (Sun D. et al., 2019). The combination of the miR-802 sponge and ultrasound microbubbles also provides a potential prevention strategy for subsequent clinical application research.

Although many miRNA targets have been tested and documented in animal models of DN, no miRNA-related biological agents are being used in the clinic, since it is still a relatively young field. We believed that in the near future, miR-related small molecules or biological agents that target specific miRNAs in DN progression will be translated into clinical studies and even clinical applications with the advantages of high targeting, accuracy and efficacy, based on the studies summarized in this review. There is certainly still some way to go due to the long-term safety and complexity.

## Concluding Remarks

DN is an inflammatory immune disorder accompanying diabetes mellitus, and studying its mechanism, exploring targets and improving therapeutic strategies have received intensive interest. Even though some drugs are available for the treatment of DN in clinical practice, they cannot achieve the desired therapeutic effect in the majority of patients. Under such a situation, new therapeutic methods and strategies are urgently needed to prevent and treat the DN. In recent years, miRNAs have aroused growing concern for their critical epigenetic modification roles in regulating gene expression and related signaling transduction of various pathological processes. Among these miRNAs, several miRNA-mediated signaling pathways are involved in the complicated inflammatory and immune processes of DN. Due to various potentially important effects of different miRNAs on the inflammatory and immune processes of DN, miRNAs are being seriously considered for attractive therapeutic targets and intervention strategies for these processes. This review illustrates the target inflammatory immune molecules, signaling transductions, target cells and detailed mechanisms based on potential target miRNAs and the important roles of these molecules to offer insights into the pathogenesis of DN and seek new treatment options for DN. After analyzing the extensive evidence, our review suggests that miRNAs have enormous therapeutic target and intervention strategy potentialities for the inflammatory and immune processes in DN. However, more substantial research is required and needed to strengthen and consolidate these research achievements and promote translational research regarding the exact functions/mechanisms of miRNAs in the inflammatory and immune processes to develop fruitful therapeutic targets and strategies for DN.

## Author Contributions

HZ and W-JN designed the ideas. W-JN helped HZ to deal with the information efficiently and draw the original figures. HZ wrote the manuscript. W-JN, X-MM, and L-QT revised the manuscript. All authors agree to be accountable for the content of the work.

## Conflict of Interest

The authors declare that the research was conducted in the absence of any commercial or financial relationships that could be construed as a potential conflict of interest.
