# Corrigendum: MicroRNAs as Regulators of Immune and Inflammatory Responses: Potential Therapeutic Targets in Diabetic Nephropathy

**DOI:** 10.3389/fcell.2022.875280

**Published:** 2022-03-01

**Authors:** Hong Zhou, Wei-Jian Ni, Xiao-Ming Meng, Li-Qin Tang

**Affiliations:** ^1^ Department of Pharmacy, Anhui Provincial Cancer Hospital, The First Affiliated Hospital of USTC, Division of Life Sciences and Medicine, University of Science and Technology of China, Hefei, China; ^2^ Inflammation and Immune Mediated Diseases Laboratory of Anhui Province, Anhui Institute of Innovative Drugs, School of Pharmacy, Anhui Medical University, Hefei, China; ^3^ Department of Pharmacy, Anhui Provincial Hospital, The First Affiliated Hospital of USTC, Division of Life Sciences and Medicine, University of Science and Technology of China, Hefei, China

**Keywords:** epigenetic regulation, miRNAs, inflammatory, cellular signal transduction, therapeutic target, diabetic nephropathy, immune

In the published article, there was an error in affiliation **1**. Instead of “Division of Life Sciences and Medicine, Department of Pharmacy, Anhui Provincial Cancer Hospital, The First Affiliated Hospital of USTC, University of Science and Technology of China, Hefei, China”, it should be “Department of Pharmacy, Anhui Provincial Cancer Hospital, The First Affiliated Hospital of USTC, Division of Life Sciences and Medicine, University of Science and Technology of China, Hefei, China”.

In the published article, there was an error in affiliation **3**. Instead of “Division of Life Sciences and Medicine, Department of Pharmacy, Anhui Provincial Hospital, The First Affiliated Hospital of USTC, University of Science and Technology of China, Hefei, China”, it should be “Department of Pharmacy, Anhui Provincial Hospital, The First Affiliated Hospital of USTC, Division of Life Sciences and Medicine, University of Science and Technology of China, Hefei, China”.

The authors apologize for this error and state that this does not change the scientific conclusions of the article in any way. The original article has been updated.

